# Effect of dual bacterial combinations on *in vitro* nutrient degradability, gas production, methane emission, ruminal fermentation parameters and predictive values in sheep

**DOI:** 10.1038/s41598-026-37458-2

**Published:** 2026-02-17

**Authors:** Ali S. A. Saleem, Sabry M. Bassiony, Sameh A. Abdelnour, Usama M. Abdel-Monem, Mohamed Y. Elaref, Khaled M. Al-Marakby

**Affiliations:** 1 Animal Production Department, Faculty of Agriculture, Sohag University, Sohag, Egypt; 2 Animal Production Department, Faculty of Agriculture, Zagazig University, Zagazig, Egypt

**Keywords:** Probiotics, Bacterial combinations, Methane emissions, Gas production, Fermentation parameters, Predictive values, Nutrients degradability, Biotechnology, Microbiology

## Abstract

The aim of the current study was to investigate the effects of incubating the tested feed with three dual combinations of two strains each at two doses: 2 × 10^9^ (low; 2) and 4 × 10^9^ (high; 4) CFU/g feed/combination on nutrient degradability, gas production, methane emission, fermentation parameters, and protozoa count using a sheep model. The three combinations were *Lactobacillus acidophilus* + *Lactobacillus bulgaricus* (AB_2_ and AB_4_*)*,* Lactobacillus casei* + *Lactobacillus plantarum* (CP_2_ and CP_4_), and *Bacillus licheniformis + Bacillus subtilis* (LS_2_ and LS_4_). The *in vitro* evaluation demonstrated a significant increase in gas production (*P* < 0.001) and a significant decrease in methane emissions (*P* < 0.001) with all probiotic combinations. Among them, AB and CP_c_ exhibited superiority compared to the control group (*P* < 0.001). The AB_2_ group had the highest dry matter content compared to CP_4_, LS_2_, LS_4_, and the control group (*P* < 0.01). Crude fiber content was highest in AB_2_, AB_4_, CP_2_, and CP_4_ compared to the other groups (*P* < 0.01). Total gas production (TGP) was improved in all probiotic groups at different time points with the best results in the AB_2_, AB_4_, CP_2_, and CP_4_ groups (*P* < 0.01). The predictive values of organic matter degradability (OMD), short-chain fatty acids (SCFA), microbial crude protein (MCP), metabolizable energy (ME), and net energy for lactation (NEL) were significantly improved in all tested probiotics, with the greatest improvement observed in the AB_2_ group (*P* < 0.001). All tested probiotics showed significantly lower values for NH_3_-N, pH, and protozoa count, while TVFA concentrations were significantly higher compared to the basal diet without probiotic supplements (*P* < 0.001). The combinations AB and CP_c_ produced the most favorable results among all the *in vitro* tested parameters. The findings demonstrate the potential of a dual probiotic strategy to enhance *in vitro* fermentation efficiency. This approach improves nutrient degradability and associated predictive values, while also reducing methane production. This research provides valuable insights for implementing sustainable and efficient dietary interventions in the sheep industry, particularly in light of the challenges posed by climate change.

## Introduction

The rapidly expanding human population poses a significant challenge to global food security, particularly in developing countries like Egypt where animal proteins are essential for nutrition. The livestock industry plays a crucial role in ensuring national food security in Egypt. With the increasing global demand for animal-derived foods and growing awareness of the environmental and health issues related to livestock production, there is a pressing need for the industry to improve productivity, animal welfare, minimize ecological impact, and adopt safer and more sustainable production practices^1,2^. One of the environmental concerns associated with ruminant livestock is enteric methane emissions, which contribute to climate change and represent an inefficiency in energy utilization, accounting for up to 12% of the gross energy lost during digestion^3^. Mitigating emissions is crucial for sustainability and production efficiency. Probiotics can reduce methane output by modulating rumen microbiota^4^. Biological feed additives, specifically probiotics, are gaining increasing attention due to their ability to modulate rumen microbiota and fermentation, leading to improved nutrient utilization and reduced methane production^5^. For instance, Abdelkarim et al. ^6^. demonstrated that a multi-strain probiotic blend significantly attenuated enteric methane emissions in sheep. Nevertheless, further investigation is required to determine the efficacy of various multi-strain formulations and to establish optimal dietary inclusion rates ^1,2^.

Antibiotics have been widely used in livestock production for treating infections and promoting growth ^7^. However, their overuse has led to the development of antimicrobial resistance in animals, posing a risk to human health. In response, the European Union banned antibiotic growth promoters in 2006. Researchers are now exploring alternatives such as probiotics to improve animal health and performance.

Probiotics are live microorganisms, have shown promise in enhancing animal health and performance^8^. Proper use of probiotics is seen as a safe and sustainable alternative to antibiotics^1,9,10^. Proper administration of probiotics is becoming a viable, safe, and sustainable strategy for replacing antibiotics^11^. Wang et al. ^12^. demonstrated that microbial inoculants can markedly influence rumen fermentation characteristics, including reducing methane production and enhancing total gas production and digestibility of feed substrates.

The integration of multiple probiotic strains with complementary functional profiles may elicit enhanced benefits through synergistic interactions that exceed the efficacy of single-strain formulations^10,13^. Within ruminant nutrition, the genera *Lactobacillus*, *Bacillus*, *Bifidobacterium*, and *Saccharomyces* constitute the primary microbial taxa utilized^14^. The biopotency of these supplements is contingent upon a complex interplay of variables, including strain specificity, dosage, administration frequency, and broader husbandry practices^2^. Furthermore, the site of action is strain-dependent; while specific microbes primarily modulate the ruminal ecosystem, others exert more pronounced effects on lower gastrointestinal function^15^.

Probiotics have shown potential to positively modulate the rumen ecosystem enhancing digestibility, reducing the methane emissions, controlling the acidosis, increasing nutrient absorption, and supporting the natural immune response mechanisms in ruminants which is reflected in animal wellbeing^2,11,16^. A recent an *in vitro* study evaluated the effects of a multi-strain probiotic (quadratic combination) blend consisting of *Lactobacillus acidophilus*, *Lactobacillus bulgaricus*, *Bacillus licheniformis*, and *Bifidobacterium bifidum* at two supplementation levels 2 × 10⁹ CFU/g and 4 × 10⁹ CFU/g administered with or without *Saccharomyces cerevisiae* (SC), on rumen fermentation parameters and nutrient degradability in sheep^10^. The findings revealed significant improvements in nutrient digestibility (IVDMD and IVCFD), reduced methane production, enhanced fermentation characteristics such as total volatile fatty acids and NH₃-N, and modulation of protozoal counts^10^. These outcomes suggest that such multi-strain combinations may offer a practical and sustainable strategy for improving ruminal function while mitigating environmental impacts.Despite these encouraging results, the precise mechanisms through which probiotics exert these effects particularly in multi-strain formulations remain poorly understood^11^. Most studies have primarily examined the impact of single-strain probiotic supplementation, with limited information on the effects of multi-strain blends, especially in *in vitro* conditions mimicking rumen fermentation.

Based on the documented efficacy of individual probiotic strains, we hypothesized that a multi-species formulation would elicit synergistic effects on ruminal metabolism. Specifically, it was posited that incorporating these probiotic blends at two inclusion levels (low: 2 × 10⁹ CFU/g feed and high: 4 × 10⁹ CFU/g feed per combination) would enhance nutrient degradability and optimize fermentation kinetics, while simultaneously suppressing methanogenesis and modulating protozoal populations. Using an *in vitro* batch culture system, this study aimed to evaluate how these microbial interactions influence gas production dynamics and predicted nutritional values, which may ultimately serve as an agent for improved animal performance.

## Materials and methods

### Location of study and ethical statement

The animal-based experiments for this study were conducted at the Animal Nutrition Research Unit, Faculty of Agriculture, Zagazig University, located in Zagazig, Egypt. All experimental procedures and protocols strictly adhered to Directive 2010/63/EU of the European Parliament and of the Council, dated September 22, 2010, which governs the protection of animals used for scientific purpose. The investigative methods were authorized by the Sohag University Scientific Research Ethics Committee, Faculty of Agriculture, Sohag University, Sohag, Egypt (Ethical code: Sohag-IACUC/6/12/1/2024/01). Furthermore, all methods, protocols, and animal handling were conducted in accordance with the ARRIVE Guidelines 2.0.

### Experimental design and probiotic combinations

A completely randomized design was used to investigate the effects of three dual combinations of multispecies bacterial strains at two levels/each on rumen fermentation parameters using the *in vitro* gas production technique. The basal diet with no probiotic supplements served as a control while the supplemented bacterial combinations added to the tested feed were *Lactobacillus acidophilus* plus *Lactobacillus bulgaricus* (AB; at a ratio of 1:1) at levels of 2 × 10^9^ (AB_2_; low) and 4 × 10^9^ (AB4; high) CFU/g of feed, *Lactobacillus casei* plus *Lactobacillus plantarum* (CP_c_; at a ratio of 1:1) at levels of 2 × 10^9^ (CP_2_; low) and 4 × 10^9^ (CP_4_; high) CFU/g of feed, and *Bacillus licheniformis* plus *Bacillus subtilis* (LS; at a ratio of 1:1) at levels of 2 × 10^9^ (LS_2_; low) and 4 × 10^9^ (LS_4_; high) CFU/g of feed. All the probiotic strain combinations were obtained from Egyptian International Pharmaceutical Industries Co (EIPICO), Tenth of Ramadan City − 1 st Industrial Zone B1, Egypt, and the preparations were in powder form consisting of the bacteria.

### Diet and proximate chemical examination

The basal diet was formulated with a 50:50 ratio of roughage to concentrate. The formulation and proximate chemical composition of the diet are presented in Table 1. The concentrate mixture and berseem hay were finely powdered (1 mm) and mixed in a 50:50 ratio before being used for chemical composition analysis and *in vitro* gas production trials. The samples were analyzed for dry matter (DM), ash, crude protein (CP), ether extract (EE), and crude fiber (CF) in the feed according to the AOAC^17^ guidelines.

### Inoculum donor and Preparation

Fresh rumen fluid was obtained from four Saidi rams that had been fed *ad libitum* on a basal diet composed of 50% concentrate and 50% berseem hay. The diet was offered twice daily at 8:00 AM and 6:00 PM for four weeks before collection to stabilize microbial activity in the rumen. On the morning of sampling, rumen contents were withdrawn by stomach tubing from each animal before the first feeding. The contents were promptly transferred into pre-warmed insulated containers and maintained under strict anaerobic conditions to preserve microbial viability. The fluid was then filtered through four layers of sterile cheesecloth and kept at 39 °C in a CO₂-enriched environment until used for *in vitro* incubation.

**Table 1:** Formulation and chemical composition of the tested diet.

Ingredients		%	
Soybean meal (44%)		7.50	
Berseem hay		50.00	
Limestone		0.60	
Wheat barn		6.50	
Mineral and vitamin mixture*		0.15	
Common salt		0.25	
Yellow corn		35.00	

Proximate chemical assays (on DM basis)			
Items	Concentrate mixture	Berseem hay	Total mixed diets (calculated)
(g/kg)			
Dry matter	889.3	912.8	901.1
Organic matter	878.4	858	868.2
Crude fibre	95.8	359	227.4
Nitrogen free extract	590.8	331.4	461.1
Crude protein	142	151	146.5
Ether extract	49.8	16.6	33.2
Ash	121.6	142	131.8

*: vitamins and minerals mixture contained, Iodine 800 mg, Cu 30,000 mg Se 300 mg, Fe 10,000 mg, MgO 80,000 mg, Zinc 100,000 mg, Cobalt 400 mg, Vit. A 10,000,000 IU, Vit. E 35,000 IU, Vit. D_3_ 2,500,000 IU, plus CaCO_3_ to 3 Kg.

### *In vitro* gas production

The *in vitro* incubation trials were conducted using a completely randomized design (CRD), involving four dietary treatments with six replicates per treatment in run. The entire protocol was repeated over three independent experimental runs, resulting in a total of 42 replicates per run. Additionally, three blank tubes (containing no substrate) were included in each run to account for background gas production originating solely from the inoculum.

The buffer medium (MB9) used for incubation was prepared to contain 0.1 g/L MgSO₄·7 H₂O, 2.8 g/L NaCl, 0.1 g/L CaCl₂, 6 g/L Na₂HPO₄, and 2 g/L KH₂PO₄·H₂O, with the pH adjusted to 6.8. To ensure anaerobic conditions, the buffer solution was saturated with CO₂ for 30 min, following the method described by Onodera and Henderson^18^. The inoculum was prepared by mixing the buffer and rumen fluid in a 2:1 (v/v) ratio. From this mixture, 30 mL aliquots were dispensed into pre-warmed 50 mL glass incubation tubes containing 200 mg of finely ground total mixed diet. The tubes were sealed with rubber stoppers equipped with tri-way valves and connected to calibrated plastic syringes for gas collection, according to the method outlined by Tilley and Terry^19^. Gas production was measured at predefined incubation intervals (3, 6, 12, 24, 36, and 48 h) using calibrated syringes. Methane concentration in the produced gas was subsequently estimated using 10 M NaOH solution, following the procedure described by Fievez et al. ^20^. At each time point, pH was immediately recorded in all tubes using a digital pH meter (Model 6010 N, Jenco Instruments Inc., San Diego, CA, USA).

### Assessment of partitioning factors, nutrient degradation, ammonia-N, volatile fatty acids concentrations, and protozoa count

Following the 48-hour incubation period, the contents of three incubation tubes from each treatment group were utilized to assess *in vitro* dry matter degradability (IVDMD). Each tube received 30 mL of neutral detergent solution, after which the mixture was thoroughly homogenized, refluxed at 105 °C for 3 h, filtered using pre-weighed Gooch crucibles, and then dried at 105 °C for another 3 h. The remaining residue was subsequently weighed to calculate IVDMD, following the method described by Blümmel and Becker^21^. Thereafter, *in vitro* crude fiber degradability (IVCFD) was determined in accordance with to AOAC^17^procedures. The remaining three tubes from each treatment were designated for measuring ammonia nitrogen (NH₃-N), total volatile fatty acids (TVFA), and protozoa count. Protozoal enumeration was conducted using direct microscopic examination as outlined by Kamra et al. ^22^, while NH₃-N concentration was quantified using Conway^23^ diffusion technique TVFA concentrations were determined following the method proposed by Warner^24^.

The predictive values for organic matter digestibility (OMD%), partitioning factor (PF), microbial crude protein (MCP; mg/g DM biomass), metabolizable energy (ME; MJ/kg DM), net energy for lactation (NEL; MJ/kg DM), and short-chain fatty acids (SCFA; mmol/200 mg DM) were determined using the following equations^25^:

$${\text{OMD }}\left( \% \right)\,=\,14.88+{\text{ }}[0.889 \times {\mathrm{GP}}]{\text{ }}+{\text{ }}{(0.45 \times {\mathrm{CP}}\left] {{\text{ }}+{\text{ }}} \right[0.0651 \times {\mathrm{XA}}]}$$

Where GP: 24 h net gas production [mL/200 mg), CP: crude protein (%), and XA: ash (%).

PF=total degradable organic matter (mg)/the produced gas^26^ in 24 h ^21^.

$${\mathrm{MCP}}\,=\,{\text{DMD }} - {\text{ }}[{\mathrm{GP}} \times 2.2]$$

Where DMD: DM degradability (mg), GP: gas production^26^, and 2.2: is a stoichiometric factor that expresses mg of C, H, and O required to produce SCFA gas associated with production of 1 mL of gas.

$${\mathrm{ME}}=[0.157 \times {\mathrm{GP}}\left] + \right[0.0084 \times {\mathrm{CP}}\left] + \right[0.022 \times {\mathrm{EE}}\left] - \right[0.0081 \times {\mathrm{CA}}]\,+\,1.06$$

$${\mathrm{NEL}}=(0.115 \times {\mathrm{GP}})+(0.0054 \times {\mathrm{CP}})+(0.014 \times {\mathrm{EE}}) - (0.0054 \times {\mathrm{CA}}) - {0.36^{}}$$

Where GP: 24 h net gas production (mL/200 mg DM), CP: crude protein (%), EE: ether extract and CA: ash (%).

$${\text{SCFA }}={\text{ }}[0.0222 \times {\mathrm{GP}}]\, - \,0.00425$$

Where GP: 24 h net gas production (mL/200 mg DM).

### **Statistical analysis**

Data analysis was achieved using IBM SPSS Software (version 27). Analysis of variance (ANOVA) was shown for all investigated parameters. The underlying statistical model is:

$${Y_{ij}}\,=\,\mu \,+\,{\alpha _i}\,+\,{e_{ij}}_{{}}$$

Where: *Y*_*ij*_ is an observation, *µ* is the mean, *αi* is the treatment effect (probiotic combination), and *e*_*ij*_ is the standard error. The significant differences among means were analyzed by Duncan’s multiple comparison test^27^.

## Results

### Effect of dual bacterial combinations on nutrients degradability

The effects of different bacterial combinations on *in vitro* nutrient degradability were present in Table 2. These combinations (AB, CP_c_, and LS) showed significantly higher values (*P* < 0.001) of IVDMD and IVCFD compared to the control. The highest values of IVDMD and IVCFD were observed in the AB_2_ (*lactobacillus acidophilus* + *Lactobacillus bulgaricus*; 2 × 10^9^), while the lowest values were obtained in the control (77.67 and 69.81% vs. 60.00 and 57.64%, respectively). There was no significant difference between the AB and CP_c_ treatments in terms of IVCFD values. The two levels (2 × 10^9^; low and 4 × 10^9^; high) within each treatment have no significant influence on the IVDMD and IVCFD. The low probiotic level was enough to improve the nutrient degradability in the current experiment.

The effect of treatment on IVDMD and IVCFD varies depending on the specific bacterial strain combinations used. All the bacterial combinations examined showed enhanced IVDMD and IVCFD values (*P* < 0.001) compared to the control, with AB_2_ having the best values among them. A low probiotic level was sufficient to improve nutrient degradability in the current experiments.

**Table 2:** Effect of dual bacterial combinations on *in vitro* nutrient degradability.

Treatments	Nutrient degradability (mg/g DM)	
	Dry matter	Crude fiber
**Control**	600.00 ± 5.77^d^	570.64 ± 4.90^c^
**AB**_**2**_	776.67 ± 4.41^a^	690.81 ± 17.40^a^
**AB**_**4**_	770.00 ± 5.77^ab^	670.92 ± 7. 50^a^
**CP**_**2**_	758.33 ± 4.41^ab^	680.30 ± 8. 70^a^
**CP**_**4**_	750.00 ± 7.64^b^	670.55 ± 7.50^a^
**LS**_**2**_	713.33 ± 4.41^c^	640.67 ± 6.70^b^
**LS**_**4**_	725.00 ± 14.43^c^	640.91 ± 6.60^b^
***P*** **-value**	0.001	0.001

AB_2_ and AB_4_ refer to dietary supplementation with *L. acidophilus* and *L. bulgaricus* at concentrations of 2 and 4 × 10⁹ CFU/g of feed, respectively. CP_2_ and CP_4_ correspond to the addition of *L. casei* and *L. plantarum* at the same respective concentrations (2 × 10⁹ and 4 × 10⁹ CFU/g). LS_2_ and LS_4_ represent the inclusion of *B. licheniformis* and *B. subtilis* at 2 and 4 × 10⁹ CFU/g of feed, respectively. ^a, b, c and d^ Within each column, means followed by different superscript letters indicate statistically significant differences at *P* < 0.05.

### Effect of dual bacterial combinations on gas production

The effects of the bacterial probiotic combinations and their levels on total gas production (mL/g DM) at different sampling times (3, 6, 12, 24, 36 and 48 h) are displayed in Table 3. It was observed that total gas production increased as the incubation time progressed across all treatments. The total gas production values with all tested probiotic combinations were significantly different from the control (*P* < 0.001). After 3 and 6 h of incubation, AB2 (*Lactobacillus acidophilus* + *L. bulgaricus*; 2 × 10^9^) exhibited the highest total gas production among the treatments, with no significant differences compared to CP_2_. Additionally, there were no significant differences in the cumulative TGP between combinations of AB (*L. acidophilus* + *L. bulgaricus*; 2 × 10^9^ and 4 × 10^9^) and CP_c_ (*L. casei* + *L. plantarum*; 2 × 10^9^ and 4 × 10^9^) at 12, 36, and 48 h. Significant differences in total gas production were observed between LS_2_ and LS_4_ at each incubation period. Overall, the lowest total gas production was recorded with the control, while the highest was seen with the low dose of AB (AB_2_) at all sampling times.

**Table 3:** Effect of dual bacterial combinations on *in vitro* total gas production.

Item	Total gas production (mL/g DM) at each sampling time hours					
	3	6	12	24	36	48
**Control**	57.10 ± 1.55^d^	93.54 ± 1.59^e^	132.10 ± 1.58^d^	164.36 ± 2.13^d^	192.90 ± 2.76^d^	198.11 ± 2.80^d^
**AB**_**2**_	76.89 ± 1.23^a^	145.00 ± 1.80^a^	191.67 ± 2.95^a^	239.18 ± 4.41^a^	272.52 ± 4.29^a^	278.77 ± 4.84^a^
**AB**_**4**_	70.83 ± 3.03^ab^	135.63 ± 3.33^bc^	185.41 ± 3.21^a^	232.49 ± 3.20^a^	262.49 ± 2.93^a^	272.08 ± 3.13^a^
**CP**_**2**_	75.02 ± 1.64^a^	138.75 ± 2.72^ab^	188.54 ± 3.77^a^	235.85 ± 4.39^a^	267.31 ± 5.24^a^	273.14 ± 5.58^a^
**CP**_**4**_	67.29 ± 4.06^bc^	133.77 ± 5.35^bc^	183.75 ± 6.88^a^	230.42 ± 6.73^a^	260.42 ± 8.05^a^	271.25 ± 8.43^a^
**LS**_**2**_	62.73 ± 1.47^c^	118.52 ± 1.90^d^	151.48 ± 2.23^c^	182.28 ± 2.16^c^	210.48 ± 2.04^c^	216.32 ± 2.06^c^
**LS**_**4**_	65.40 ± 1.64^b^	128.14 ± 2.31^c^	171.46 ± 2.80^b^	216.04 ± 3.14^b^	245.83 ± 4.08^b^	252.71 ± 3.56^b^
***P*** **-value**	0.001	0.001	0.001	0.001	0.001	0.001

AB_2_ and AB_4_ refer to dietary supplementation with *L. acidophilus* and *L. bulgaricus* at concentrations of 2 and 4 × 10⁹ CFU/g of feed, respectively. CP_2_ and CP_4_ correspond to the addition of *L. casei* and *L. plantarum* at the same respective concentrations (2 × 10⁹ and 4 × 10⁹ CFU/g). LS_2_ and LS_4_ represent the inclusion of *B. licheniformis* and *B. subtilis* at 2 and 4 × 10⁹ CFU/g of feed, respectively. ^a, b, c, d^ Within each column, means followed by different superscript letters indicate statistically significant differences at *P* < 0.05.

### Effect of dual bacterial combinations on methane emission

Data presented in Table 4 shows the effect of different bacterial combinations on methane (CH_4_) production at various measuring times (3, 6, 12, 24, 36, and 48 h). The control group consistently showed the highest CH_4_ production (*P* < 0.001) across all incubation times compared to the probiotic combinations (AB, CP_c_, and LS). Among the treatments, AB_4_ (*Lactobacillus* acidophilus + *Lactobacillus* bulgaricus; 4 × 10^9^ CFU/g) exhibited the lowest release of CH_4_ values at all incubation times. There were no statistical variations in CH_4_ emissions between the low and high doses of all the tested probiotic combinations during the incubation period. This suggests that these probiotics may have a mitigating effect on CH_4_, possibly by altering microbial populations or fermentation pathways. Lower CH_4_ emissions are desirable as they indicate more efficient energy utilization by the host animal.

**Table 4:** Effect of dual bacterial combinations on methane emission.

Item	Methane emission (mL/g DM) at different hours					
	3	6	12	24	36	48
**Control**	37.08 ± 1.89^c^	52.07 ± 1.01^c^	64.81 ± 1.80^c^	72.07 ± 2.24^d^	82.07^d^ ±2.88^d^	82.07 ± 2.88^d^
**AB**_**2**_	12.73 ± 1.87^a^	23.33 ± 2.18^a^	27.48 ± 2.75^a^	31.65 ± 2.99^ab^	33.73 ± 2.81^ab^	33.73 ± 2.81^ab^
**AB**_**4**_	11.06 ± 0.94^a^	18.54 ± 1.14^a^	24.15 ± 1.51^a^	24.57 ± 2.39^a^	25.40 ± 2.04^a^	25.40 ± 2.04^a^
**CP**_**2**_	16.67 ± 1.98^b^	25.00 ± 2.81^a^	29.57 ± 3.40^a^	38.73 ± 3.63^b^	42.48 ± 3.94^b^	42.48 ± 3.94^b^
**CP**_**4**_	15.83 ± 0.77^b^	20.00 ± 1.96^a^	27.69 ± 2.53^a^	33.53 ± 2.79^ab^	38.94 ± 3.28^b^	38.94 ± 3.28^b^
**LS**_**2**_	28.75 ± 1.71^c^	40.00 ± 2.04^b^	49.98 ± 2.11^b^	58.73 ± 2.51^c^	66.86 ± 2.88^c^	66.86 ± 2.88^c^
**LS**_**4**_	29.60 ± 1.41^c^	42.71 ± 1.95^b^	52.73 ± 1.82^b^	60.63 ± 1.85^c^	67.50 ± 2.14^c^	67.50 ± 2.14^c^
***P*** **-value**	0.001	0.001	0.001	0.001	0.001	0.001

AB_2_ and AB_4_ refer to dietary supplementation with *L. acidophilus* and *L. bulgaricus* at concentrations of 2 and 4 × 10⁹ CFU/g of feed, respectively. CP_2_ and CP_4_ correspond to the addition of *L. casei* and *L. plantarum* at the same respective concentrations (2 × 10⁹ and 4 × 10⁹ CFU/g). LS_2_ and LS_4_ represent the inclusion of *B. licheniformis* and *B. subtilis* at 2 and 4 × 10⁹ CFU/g of feed, respectively. ^a, b, c and d^ Within each column, means followed by different superscript letters indicate statistically significant differences at *P* < 0.05.

### Effect of dual bacterial combinations on fermentation parameters and protozoa counts

**Fig. 1:**
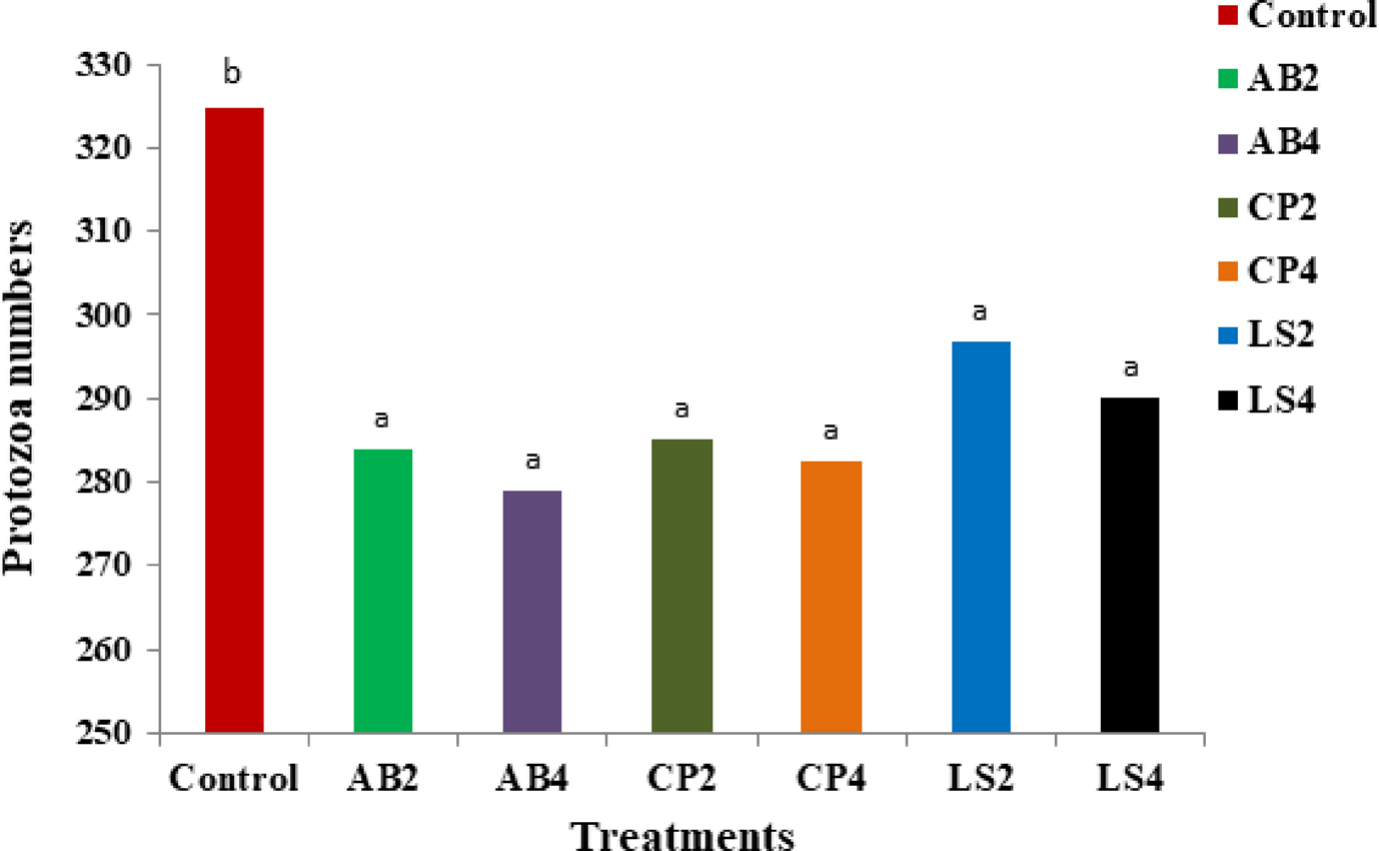
Effect of dual bacterial combinations on protozoa counts. AB_2_ and AB_4_ refer to dietary supplementation with *L. acidophilus* and *L. bulgaricus* at concentrations of 2 and 4 × 10⁹ CFU/g of feed, respectively. CP_2_ and CP_4_ correspond to the addition of *L. casei* and *L. plantarum* at the same respective concentrations (2 × 10⁹ and 4 × 10⁹ CFU/g). LS_2_ and LS_4_ represent the inclusion of *B. licheniformis* and *B. subtilis* at 2 and 4 × 10⁹ CFU/g of feed, respectively. ^a, b, c and d^ Within each column, means followed by different superscript letters indicate statistically significant differences at *P* < 0.05.

The fermentation parameters, including NH_3_-N, TVFA, pH, and protozoa count with the assessed probiotic combinations are presented in Table 5; Fig. 1, respectively.

The concentrations of NH_3_-N, pH values, and protozoa counts were significantly (*P* < 0.001) reduced by the tested probiotic treatments (AB, CP_c_, and LS), while the TVFA concentrations were significantly (*P* < 0.001) elevated compared to the control group. Among the tested probiotic combinations, the lowest values of NH_3_-N, pH, and protozoa count (26.46, 5.66, and 279, respectively) were observed with AB_4_ (high AB dose), with no statistical differences compared to AB_2_ (low AB dose). Additionally, the TVFA concentrations showed an opposite trend in the AB treatments. There were no considerable effects of the two tested doses (low and high) within each probiotic combination on the fermentation measurements and protozoa count (Fig. 1). All the probiotic blends (AB, CP_c_, and LS) have a comparable impact on protozoa counts.

**Table 5:** Effect of dual bacterial combinations on fermentation parameters such as NH_3_-N, TVFA and pH.

Item	Fermentation parameters		
	NH_3_-*N* (mg/dL)	TVFA (ml eq/L)	pH
**Control**	38.08 ± 1.12^d^	190.00 ± 4.04^c^	5.90 ± 0.04^a^
**AB**_**2**_	28.98 ± 1.26^a^	211.00 ± 3.21^ab^	5.70 ± 0.01^cd^
**AB**_**4**_	26.46 ± 2.18^a^	215.67 ± 2.4^a^	5.66 ± 0.02^cd^
**CP**_**2**_	32.76 ± 1.26^bc^	204.00 ± 4.04^ab^	5.79 ± 0.04^bc^
**CP**_**4**_	31.50 ± 1.26^bc^	207.00 ± 3.21^ab^	5.76 ± 0.04^bc^
**LS**_**2**_	34.72 ± 1.12^cd^	200.67 ± 8.95^bc^	5.84 ± 0.03^ab^
**LS**_**4**_	35.84 ± 1.12^cd^	196.00 ± 3.79^bc^	5.80 ± 0.06^ab^
***P*** **-value**	0.001	0.001	0.001

AB_2_ and AB_4_ refer to dietary supplementation with *L. acidophilus* and *L. bulgaricus* at concentrations of 2 and 4 × 10⁹ CFU/g of feed, respectively. CP_2_ and CP_4_ correspond to the addition of *L. casei* and *L. plantarum* at the same respective concentrations (2 × 10⁹ and 4 × 10⁹ CFU/g). LS_2_ and LS_4_ represent the inclusion of *B. licheniformis* and *B. subtilis* at 2 and 4 × 10⁹ CFU/g of feed, respectively. ^a, b, c and d^ Within each column, means followed by different superscript letters indicate statistically significant differences at *P* < 0.05.

### Effect of dual bacterial combinations on predictive values of organic matter degradability, short chain fatty acids, partitioning factor, microbial crude protein, metabolizable energy, and net energy for lactation

Table 6 shows the effects of bacterial probiotic mixtures on the predictive values of OMD, SCFA, PF, MCP, ME, and NEL. The probiotic supplements AB, CP_c_, and LS showed higher values of OMD, SCFA, MCP, ME, and NEL, while PF had an opposite tendency compared to the control. Both high and low levels of the combinations AB and CP_c_ showed no significant differences in the predictive values mentioned, and the highest values were recorded with AB_2_. However, except for the predictive values of MCP, there were no statistical variations between LS_2_ and LS_4_.

**Table 6:** Effect of dual bacterial combinations on predictive values of organic matter degradability, short chain fatty acids, partitioning factor, microbial crude protein, metabolizable energy, and net energy for lactation.

Item	Predictive values					
	SCFA Mmol	ME MJ/kg DM	NEL MJ/kg DM	OMD %	MCP mg/g DM	PF mg TOMD/mL gas
**Control**	0.73 ± 0.01^d^	6.31 ± 0.07^d^	3.48 ± 0.05^d^	51.09 ± 0.38^d^	525.94 ± 5.93^d^	1.56 ± 0.01^d^
**AB**_**2**_	1.06 ± 0.02^a^	8.66 ± 0.14^a^	5.20 ± 0.1^a^	64.40 ± 0.78^a^	667.76 ± 3.71^a^	1.35 ± 0.01^a^
**AB**_**4**_	1.03 ± 0.01^a^	8.45 ± 0.1^a^	5.04 ± 0.07^a^	63.21 ± 0.57^a^	665.32 ± 6.42^a^	1.36 ± 0.01^a^
**CP**_**2**_	1.04 ± 0.02^a^	8.56 ± 0.14^a^	5.12 ± 0.1^a^	63.81 ± 0.78^a^	653.46 ± 4.32^ab^	1.35 ± 0.01^a^
**CP**_**4**_	1.02 ± 0.03^a^	8.38 ± 0.21^a^	5.00 ± 0.15^a^	62.84 ± 1.2^a^	648.80 ± 8.24^abc^	1.37 ± 0.01^a^
**LS**_**2**_	0.81 ± 0.01^c^	6.87 ± 0.07^c^	3.89 ± 0.05^c^	54.28 ± 0.38^c^	631.20 ± 4.97^bc^	1.49 ± 0.01^c^
**LS**_**4**_	0.95 ± 0.01^b^	7.94 ± 0.1^b^	4.66 ± 0.07^b^	60.28 ± 0.56^b^	628.20 ± 14.76^c^	1.40 ± 0.01^b^
***P*** **-value**	0.001	0.001	0.001	0.001	0.001	0.001

AB_2_ and AB_4_ refer to dietary supplementation with *L. acidophilus* and *L. bulgaricus* at concentrations of 2 and 4 × 10⁹ CFU/g of feed, respectively. CP_2_ and CP_4_ correspond to the addition of *L. casei* and *L. plantarum* at the same respective concentrations (2 × 10⁹ and 4 × 10⁹ CFU/g). LS_2_ and LS_4_ represent the inclusion of *B. licheniformis* and *B. subtilis* at 2 and 4 × 10⁹ CFU/g of feed, respectively. ^a, b, c and d^ Within each column, means followed by different superscript letters indicate statistically significant differences at *P* < 0.05.

## Discussion

The study evaluated the *in vitro* effects of dual probiotic bacterial combinations on nutrient degradability, gas production, methane emission, ruminal fermentation parameters, and predictive energy values. The results indicated that all probiotic treatments, particularly AB_2_, significantly enhanced nutrient utilization and fermentation efficiency, while reducing methane production. These findings support the initial hypothesis that multispecies probiotics can synergistically improve ruminal fermentation.

The increase in IVDMD values highlights the enhanced microbial efficiency in degrading feed substrates within the fermentation environment. The greatest values of IVDMD and IVCFD were detected with AB_2_ (*L. acidophilus* plus *L. bulgaricus*, 2 × 10^9^CFU/g). Similar results were reported by Sheikh et al.^28^. who found that supplementing a probiotic (*including Lactobacillus acidophilus*; 6 × 10^9^ CFU/g) to the feed (concentrate mixture and rice straw; 50:50) led to significant improvements in IVDMD, IVOMD, and IVNDFD (*P* < 0.05) after 48 h of incubation. LAB enhance cellulose digestibility by secreting ferulate esterase, an enzyme that converts ferulic acid into arabinoxylan^2,29^. Arabinoxylan, in turn, aids in the action of fiber-degrading enzymes by enhancing access through the lignin-cellulose matrix of the plant cell wall^30^.

Enhanced substrate degradability significantly modulates microbial metabolic activity and gas production kinetics, both of which serve as pivotal indicators of fermentation efficiency. The *in vitro* gas production technique remains a robust methodology for assessing feed fermentability, offering critical insights into the digestive dynamics and degradability within the ruminal environment^31,32^. Throughout the sampling times, the highest TGP were noted with the low dose (AB_2_; 2 × 10^9^ CFU/g) of AB (*Lactobacillus acidophilus* plus *Lactobacillus bulgaricus*). These results are in harmony with Jeyanathan et al.^33^. who found that probiotic at 3 × 10^10^ CFU/day of *Lactobacillus bulgaricus* D1 (*P*< 0.05) enhanced the TGP and Chen et al.^34^, who mentioned that *L. acidophilus* improved the *in vitro* gas production. In this study the best values of IVDMD and IVCFD were recorded for the AB_2_ treatment (Table 2) which aligns with Theodorou et al. ^35^, who suggested that TGP tends to increase with substrate degradation, indicating a positive correlation between feed degradation extent and cumulative gas output under *in vitro* conditions.

Given the increased gas production observed, it is essential to consider its environmental implications, particularly regarding methane emissions, a major concern in ruminant nutrition. The *in vitro* evaluation indicated that the examined dual probiotic combinations reduced methanogenesis compared to the basal diet, and AB_4_ exhibited the lowest number of CH_4_releases at all incubation times. These results are consistent with Jeyanathan et al.^33^. who mentioned that the *in vitro* CH_4_ production of several bacterial strains indicated that CH_4_ production varied with bacterial strain. They also reported that *Lactobacillus bulgaricus* (10^8^ CFU/mL) had a great impact on the *in vitro* CH_4_ production, while the same strain had no impact on the in vivo CH_4_production. In contrast, Chen, et al^34^. found that the supplementation of *L. acidophilus* at levels 0, 0.25 × 10^7^, 0.50 × 10^7^, and 0.75 × 10^7^ (CFU/mL) have not any significant impact on the *in vitro* production of CH_4_. The inconsistent results in production CH_4_ between the former authors could be attributed to the differences in fermented carbohydrates^36^, fiber fractions contents^37,38^, strains and species microbial, animal species, dosage, physiological states, and feeding regimens^2^, and probiotic strain and fermentation media^33^. Moreover, Doyle et al.^39^. stated that LAB or their metabolites could influence ruminal methanogenesis in three possible ways: (1) shifting rumen fermentation and decreasing CH_4_ release, (2) inhibiting rumen methanogens directly, and (3) inhibiting specific rumen bacteria that produce H_2_ or methyl-containing compounds that are substrates for methanogenesis.

The study examined the impact of probiotic combinations on rumen fermentation in an *in vitro* model. The addition of AB significantly enhanced fermentation efficiency and microbial activity compared to other treatments. Volatile fatty acids (VFAs) are important energy sources for ruminants and serve as indicators of ruminal digestion and metabolic activity. Carbohydrates in the diet are metabolized by rumen microbes into VFAs. The tested bacterial combinations increased TVFA concentrations in the study.

The present results are consistent with Saleem et al.^10^. who found that the concentrations of TVFA were significantly elevated (*P*< 0.05) with both supplements ABLB2 and ABLB2 + SC, respectively, compared to the basal diet before feeding and post-feeding at 3 h in sheep. Similarly, Miguel et al.^40^. demonstrated that the *in vitro* TVFA concentrations were significantly higher (*P*< 0.05) than those in the basal diet after 24 h of incubating Lactobacillus acidophilus. Additionally, Abdelbagi et al.^41^. reported that *L. plantarum* (10 strains; 0.5 mL; 10^11^ CFU/mL) resulted in a significant (*P* < 0.01) increase in TVFA levels compared to the control group after 72 h of incubation. At all sampling times, the TGP with the dual probiotic combinations, especially AB and CPc, was significantly better (*P* < 0.001) than the other treatments. These findings reflect the higher production of TVFA in the AB and CP_c_ treatments. A study^42^ also noted a strong relationship between *in vitro* TVFA production and TGP.

Regarding NH_3_-N, a decrease in concentration was observed with all tested dual combinations compared to the control. Concerning the NH_3_-N, the reduction in NH_3_-N concentration was noticed with all tested dual combinations in comparison with the control. Contreras-Govea et al.^43^. found that treating silages with *L. plantarum* resulted in lower NH_3_-N concentrations and increased *in vitro* microbial growth. Additionally, Arioli et al^44^. suggested that the urease activity of *Streptococcus thermophilus* enhances *L. bulgaricus* homolactic fermentation, potentially reducing rumen NH_3_-N concentration. _3_. A similar trend was observed by Sheikh et al.^28^. when incubating the probiotic *L. acidophilus* with the basal diet for 48 h, leading to a decrease in NH_3_-N concentration compared to the control. In addition, Saleem et al^10^. reported that the group supplemented with a lower dose (2 × 10⁹ CFU/g) of the quadratic probiotic bacteria combined with *S. cerevisiae* exhibited the lowest NH₃-N concentrations during the *in vivo* trial. The reduction in NH_3_-N levels in the present study may be attributed to the incorporation of more NH_3_-N in microbial protein synthesis^2,45^.

An inadequate concentration of NH₃-N can limit microbial protein synthesis, whereas excessive levels may impair its effective utilization by rumen microbes^46^. Therefore, the concentration of NH₃-N in the rumen serves as an indicator of the balance between protein degradation and microbial synthesis under defined dietary conditions^34^.

Ruminal pH is a crucial indicator of the internal balance in the rumen environment. Maintaining a stable pH is essential for optimizing fermentation processes and improving nutrient digestibility^47^. The results obtained for NH_3_-N and VFA were reflected in the pH values. Ruminants have well-regulated physiological mechanisms to stabilize ruminal pH within an optimal range, typically between 5.5 and 7.0, to support microbial activity and fermentation processes effectively The AB and CP_c_ dual probiotic combinations resulted in slight but statistically significant drops in pH values compared to the control and LS treatment. However, no statistically significant differences were observed in ruminal pH values with the probiotic combination ABLB^10^. Similar results were reported by Miguel et al.^40^, who found that pH values of rumen fluid decreased significantly (*P* < 0.05) after 24 h of *in vitro* fermentation with a probiotic containing Lactobacillus acidophilus.

The impact of microbial feed additives on pH has been explained by a variety of mechanisms: the competition for glucose utilization with *S. bovis* and other *Lactobacillus* species^48^, stimulation of lactic acid utilizing bacteria (LUB) ^49^, and modification of protozoa in the rumen ^50^ that compete with LAB for glucose uptake. Rapid fermentation of fermentable foods can cause significant rumen conditions, including a decrease in pH and an increase in lactic acid levels, both of which are factors in metabolic acidosis ^51^. The bacterium’s inclination to produce organic acids may be the cause of the tendency to decrease pH after the probiotic treatment.

In the present study, protozoa count significantly decreased with the supplementation of the dual probiotic combinations relative to the control. Analogous findings were noticed by Astuti et al. ^52^. who reported that the protozoa counts was)significantly decrease in the rumen fluid of the cattle for all levels of 14 strains of *L. plantarum* (1.8 × 10^10^CFU/mL) with the basal diet (concentrate: elephant grass; 70: 30%). However, Jeyanathan et al. ^33^. with *L. bulgaricus* (3 × 10^10^CFU) and Pongsub et al.^53^. with *L. casei* TH14 (10^11^CFU/g) did not find any change in the number of protozoa. Puniya et al. ^15^. reviewed that the discrepancy in probiotic action may be due to different selected strains, dosages, frequencies, and times of supplementation. During rumen fermentation, certain protozoa contribute to methane formation by producing hydrogen (H₂) as a metabolic byproduct, primarily through pathways that generate acetic acid ^54^. Reducing the protozoa count is advantageous for both the animals and the environment. Additionally, *L. plantarum*, a probiotic, has demonstrated the ability to reduce methane emissions indirectly by inhibiting protozoa, which reduces the availability of hydrogen (H₂) needed for methanogenesis. Furthermore, LAB or their bioactive metabolites, such as bacteriocins, may directly inhibit methanogenic archaea or microbes involved in hydrogen and methyl donation, disrupting the methanogenesis process and potentially reducing methane production^39,55,56^.

Modulation of fermentation profiles is expected to influence energy-related parameters in the rumen, leading to predictive values of digestibility and energy utilization. The results suggest that probiotic treatments have a positive impact on the energy and digestibility of feed items, indicating better fermentation and potentially higher nutrient availability. The tested probiotic combinations AB and CP_c_ show significantly better predictive values of OMD, SCFA, MCP, NEL, and ME than the other treatments (*P* < 0.001), with the highest values recorded with AB2.

In relation to OMD, our findings are consistent with those of Cai et al. ^57^. who discovered that a probiotic supplement (*Clostridium butyricum*) notably enhanced OMD. Probiotic therapy can offer animals amino acids, SCFA, and vitamin B, which could influence nutrient absorption. Furthermore, it may provide a variety of digestive enzymes, including lipase, protease, and amylase, that could enhance nutrient absorption^57,58^.

The PF is the ratio of truly digested OM (TDOM) to the volume of gas (in mL) generated during *in vitro* fermentation at the time corresponding to half of the asymptotic gas production level^21^. Izuddin et al. ^59^ revealed that increasing the substrate digestibility and fiber-degrading microbe activity are indicated by raising the gas production. The TGP typically rises in parallel with the extent of substrate disappearance, reflecting a direct and positive association between feed degradation and volume of gas generated during *in vitro* fermentation trials^35^. Results are in the same line with the findings mentioned above, whereas the *in vitro* nutrient degradability and TGP were superior to AB_2_.

The results of SCFA in this trial are consistent with Paengkoum, et al^60^. who stated that the probiotic involving *Lactobacillus acidophilus* (10^10^ CFU/g) increased SCFA levels. Bergman^61^ and Van Soest^62^ indicated that SCFA are produced from the ruminal bacteria fermentation of plant fibers. Short-chain fatty acids (SCFA) contribute nearly 70% of the total energy needs in ruminants. According to Van Soest^62^and Bannink et al.^63^, the primary SCFA produced in the rumen acetate, propionate, and butyrate constitute approximately 95% of the total SCFA pool and play a critical role in supporting rumen development and functionality. Probiotic can regulate rumen fermentation and energy efficiency through altering the SCFA concentration^64^. The present investigation showed that probiotic supplementation increased SCFA levels, which is reflected in the values of ME and NEL.

The findings of the MCP were consistent with Saleem et al. ^10^, who found that the MCP content was higher when a quadratic probiotics combination, including *Lactobacillus bulgaricus*,* L. acidophilus*,* B. bifidum*, and *B. licheniformis* at two concentrations: 2–4 × 10⁹ CFU/g (ABLB2) and (ABLB4), was added to the basal diet. Similarly, supplementation with live *B. subtilis* natto at a concentration of 10⁹ CFU significantly increased MCP levels after 12 h of the *in vitro* fermentation using dairy cow rations compared to the control group. After 24 h of fermentation, *Bacillus subtilis*resulted in a 41.46% enhancement in MCP production relative to the control group. Liu et al. ^65^.concluded that SCFA can enhance nitrogen utilization in the rumen.

## Conclusion

This research highlights a promising strategy for mitigating climate change caused by ruminants: utilizing a probiotic mixture to enhance sheep industry production and sustainability while simultaneously reducing methane emissions. This *in vitro* study demonstrates that the adding of dual probiotic combinations (*Lactobacillus acidophilus* + *Lactobacillus bulgaricus* (AB_2_ and AB_4_*)*,* Lactobacillus casei* + Lactobacillus plantarum (CP_2_ and CP_4_), and *Bacillus licheniformis + Bacillus subtilis* (LS_2_ and LS_4_) in ruminant diets can significantly enhance rumen fermentation, improve nutrient utilization, reduce methane emissions, and create a more favorable rumen environment. Under the current experimental conditions, the low levels of the assessed probiotic combinations were sufficient to improve the parameters investigated. These findings strongly suggest that probiotic combinations offer a viable and sustainable alternative to antibiotics in livestock feed. The synergistic effects observed within these multi-species blends highlight their potential to significantly reduce the environmental impact of livestock production while simultaneously improving animal health and productivity. While *in vitro* findings provide a promising foundation, in vivo validation in sheep is essential to establish definitive evidence of probiotic efficacy in mitigating enteric methane emissions and contributing to broader climate change strategies. Future research should leverage high-throughput “omics” technologies (e.g., metagenomics and metabolomics) to characterize shifts in the ruminal and intestinal microbiota following probiotic administration. Such approaches will provide mechanistic insights into the complex interactions governing host health, nutrient bioavailability, and the biological pathways underlying methane abatement.

## Data Availability

The data supporting the findings of this study will be made available upon reasonable request to the corresponding author.
